# Inhibition of pro-HGF activation by SRI31215, a novel approach to block oncogenic HGF/MET signaling

**DOI:** 10.18632/oncotarget.8785

**Published:** 2016-04-18

**Authors:** Benjamin Y. Owusu, Namita Bansal, Phanindra K.M. Venukadasula, Larry J. Ross, Troy E. Messick, Sanjay Goel, Robert A. Galemmo, Lidija Klampfer

**Affiliations:** ^1^ Department of Oncology, Drug Discovery Division, Southern Research, Birmingham, AL, USA; ^2^ Department of Chemistry, Drug Discovery Division, Southern Research, Birmingham, AL, USA; ^3^ High Throughput Screening, Southern Research, Drug Discovery Division, Birmingham, AL, USA; ^4^ The Wistar Institute, Southern Research, Philadelphia, PA, USA; ^5^ Albert Einstein Cancer Center, Southern Research, Bronx, NY, USA

**Keywords:** HGF, colon cancer, EGFR

## Abstract

The binding of hepatocyte growth factor (HGF) to its receptor MET activates a signaling cascade that promotes cell survival, proliferation, cell scattering, migration and invasion of malignant cells. HGF is secreted by cancer cells or by tumor-associated fibroblasts as pro-HGF, an inactive precursor. A key step in the regulation of HGF/MET signaling is proteolytic processing of pro-HGF to its active form by one of the three serine proteases, matriptase, hepsin or HGF activator (HGFA).

We developed SRI 31215, a small molecule that acts as a triplex inhibitor of matriptase, hepsin and HGFA and mimics the activity of HAI-1/2, endogenous inhibitors of HGF activation. We demonstrated that SRI 31215 inhibits fibroblast-induced MET activation, epithelial-mesenchymal transition and migration of cancer cells. SRI 31215 overcomes primary resistance to cetuximab and gefitinib in HGF-producing colon cancer cells and prevents fibroblast-mediated resistance to EGFR inhibitors. Thus, SRI 31215 blocks signaling between cancer cells and fibroblasts and inhibits the tumor-promoting activity of cancer-associated fibroblasts.

Aberrant HGF/MET signaling supports cell survival, proliferation, angiogenesis, invasion and metastatic spread of cancer cells, establishing HGF and MET as valid therapeutic targets. Our data demonstrate that inhibitors of HGF activation, such as SRI 31215, merit investigation as potential therapeutics in tumors that are addicted to HGF/MET signaling. The findings reported here also indicate that inhibitors of HGF activation overcome primary and acquired resistance to anti-EGFR therapy, providing a rationale for concurrent inhibition of EGFR and HGF to prevent therapeutic resistance and to improve the outcome of cancer patients.

## INTRODUCTION

Hepatocyte growth factor (HGF) was identified as a mitogenic factor for hepatocytes that can promote motility and scattering of epithelial cells [1, 2]. The binding of HGF to its receptor MET activates signaling cascade which promotes the growth and survival of cancer cells and stimulates epithelial to mesenchymal transition (EMT), one of the early stages of metastatic spread [3]. Accordingly, constitutive activation of the HGF/MET signaling pathway is associated with tumor aggressiveness, resistance to therapy and predicts poor outcome in many cancers patients [4]. MET activation promotes the cancer stem cell phenotype [5, 6] and HGF/MET signaling plays a crucial role in the development of resistance to classical cytotoxic therapy and targeted therapy, such as EGFR and BRAF inhibitors [7–9]. Cancer cells with amplified MET, which normally display HGF-independent MET activation, become dependent on HGF when MET kinase activity is inhibited [10], suggesting that HGF may also be associated with resistance to drugs that target MET.

MET mutations (or MET amplification /overexpression) which trigger ligand- independent activation of signaling, are relatively rare in human cancer and occur in approximately 6% of colon cancers [11]. In contrast, HGF has been recently shown to be produced in a relatively large subset (~30%) of primary colon tumors and established colon cancer cell lines due to mutations in the HGF promoter region [12]. HGF-producing cancer cells display autocrine activation of MET signaling [13]. The levels of HGF are increased in serum and in tumor tissues in colon cancer patients, particularly in patients with lymph node and liver metastasis [14], and are associated with poor survival of stage II and stage III colon cancer patients [15]. Elevated levels of HGF are also associated with lymph node metastasis and relapse in breast cancer patients [16, 17], multiple myeloma patients [18] and myeloid leukemia patients [19].

HGF is secreted by tumor cells [12, 20, 21] or, more commonly, by tumor-associated fibroblasts [22] as pro-HGF, the inactive precursor. Proteolytic conversion of pro-HGF to its active form is the rate-limiting step in the HGF/MET signaling pathway. The trypsin-like serine proteases, matriptase, hepsin and HGFA are the principal proteases required for HGF activation [23–30]. These enzymes cleave pro-HGF to HGF 10^2^- 10^4^ times more efficiently than, for example, TMPRSS13 or uPA (urokinase plasminogen activator) [30, 31]. The activity of matriptase, HGFA and hepsin is controlled by the endogenous inhibitors of pro-HGF activation, the HGFA inhibitors HAI-1/2 [30, 32, 33]. The HGF-activating proteases are upregulated and the levels of HAI-1/2 are reduced in cancer tissues, resulting in increased activation of HGF and constitutive stimulation of HGF/MET signaling [30]. Intestinal deletion of endogenous HAI-1 augments Wnt signaling in *Apc/^Min/+^* mice, both in tumors and in normal mucosa and enhances intestinal tumor formation [34], suggesting that HAI-1 has tumor suppressor properties. Accordingly, reduced expression of the HAIs is associated with advanced disease and poor outcome in cancer patients [34–40].

We synthesized SRI 31215, a small molecule which inhibits matriptase, hepsin, and HGFA, blocks pro-HGF activation and thus mimics the activity of HAI-1/2. Cancer cells, including cell lines used in this study [41–43], commonly overexpress a combination of pro-HGF-activating proteases. Thus, triplex inhibitors, such as SRI 31215, will efficiently interfere with activation of pro-HGF in cancer cells that display expression/activation of multiple proteases. We have shown that SRI 31215 blocks signaling between colon cancer cells and fibroblasts, prevents fibroblast-dependent growth and migration of cancer cells and overcomes fibroblast-induced resistance to inhibitors of EGFR.

## RESULTS

### SRI 31215, a novel triplex inhibitor of matriptase, hepsin and HGFA, prevents HGF activation

**Figure 1:**
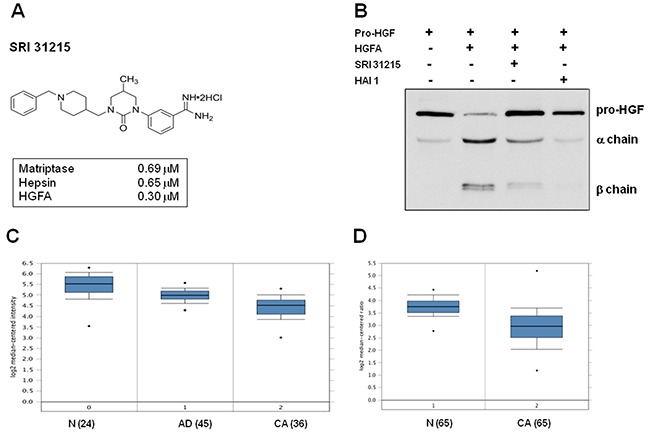
SRI 31215 inhibits the proteolytic activation of pro-HGF **A.** The structure of SRI 31215 with the IC_50_ for matriptase, hepsin and HGFA indicated. **B.** pro-HGF was incubated with activated HGFA in the absence or the presence of SRI 31215 (10 μM) or HAI-1 (20 nM) as indicated. The processing of pro-HGF was monitored by immunoblotting, using an antibody that recognizes pro-HGF as well as the α and β chains. **C.** and **D.** Oncomine analysis of HAI-1 expression in colon cancer patients as reported by Skrzypzak et al [68] (C) and Gaedcke et al [69] (D). N= normal mucosa, AD: adenoma, CA: carcinoma. The number of patients is indicated in the brackets.

We have developed a series of phenylamidine cyclic urea analogs that have inhibitory activity for matriptase, hepsin and HGFA, the three serine proteases that carry out the proteolytic activation of pro-HGF to HGF. The design of SRI 31215 (Figure 1A) was based upon a structural template adapted from inhibitors of clotting factor Xa [44, 45]. Details of the structure-based design effort have been reported elsewhere [46]. We demonstrated that SRI 31215 is an equipotent inhibitor of matriptase (IC_50_ = 0.69 μM), hepsin (IC_50_ = 0.65 μM) and HGFA (IC_50_ = 0.3 μM) (Figure 1A). While the selectivity of SRI 31215 for trypsin and thrombin is acceptable, currently we are optimizing its selectivity over factor Xa [46].

To confirm that SRI 31215 inhibits activation of pro-HGF to its biologically active form, we incubated recombinant pro-HGF with HGFA in the absence or presence of SRI 31215. Recombinant HAI-1 served as a positive control. As shown in Figure 1B, HGFA-induced cleavage of pro-HGF into alpha and beta chains was inhibited by both SRI 31215 and HAI-1.

The levels of endogenous inhibitors of HGF activation, HAI-1, are reduced in colon cancer tissues compared to normal mucosa (Figure 1C and 1D). SRI 31215 inhibits matriptase, hepsin and HGFA, prevents pro-HGF activation and therefore mimics the activity of HAI-1. As such, it may help to restore homeostasis in tissues with upregulated pro-HGF-activating machinery.

### SRI 31215 inhibits fibroblast-induced HGF/MET signaling in tumor cells

Although pro-HGF binds to the MET receptor, it does not induce MET signaling [47] and therefore lacks biological activity. We used conditioned media from 18Co and WI38 fibroblasts as a source of pro-HGF [48]. In WI38 fibroblasts HGF is detected as a single band ~90 kD, corresponding to its pro-form (Supplementary Figure S1A), consistent with published results [13]. Although WI38 cells express MET [13], these cells do not display active HGF/MET signaling, indicating that fibroblasts do not possess the proteolytic machinery that would activate pro-HGF and trigger autocrine HGF/MET signaling (Supplementary Figure S1A).

Here we show that like recombinant HGF, fibroblast-derived factors stimulate proliferation of DiFi cells (Supplementary Figure 1B). The MET kinase inhibitor JNJ 38877605 prevented both HGF- and fibroblast- induced proliferation of DiFi cells (Supplementary Figure S1B). Consistent with its mode of action, SRI 31215 did not influence proliferation induced by recombinant, active HGF, but was as efficient as JNJ 38877605 in preventing fibroblast-induced proliferation of DiFi cells (Supplementary Figure S1B).

**Figure 2:**
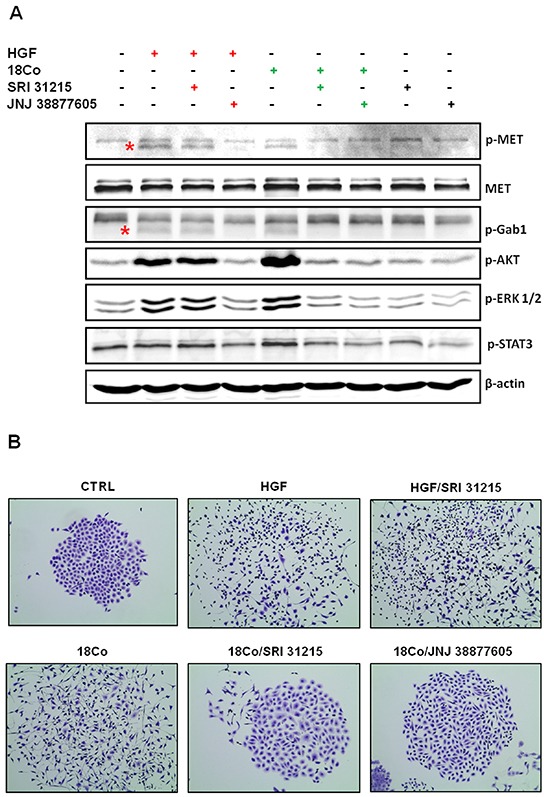
SRI 31215 inhibits the crosstalk between tumor cells and fibroblasts **A.** Inhibition of fibroblast-induced oncogenic signaling in tumor cells by SRI 31215. Serum-starved DU145 cells were treated with recombinant HGF or conditioned media from 18Co cells alone or in the presence of SRI 31215 (10 μM) or JNJ 38877605 (1 μM) for 30 minutes. The levels of pMET, pGab1, pAKT, pERK, and pSTAT3 were determined by immunoblotting. Specific bands for pMET and pGab1 are indicated by asterisks. **B.** Inhibition of fibroblast-induced cell scattering by SRI 31215. Scattering of DU145 cells was induced with recombinant HGF or with conditioned media from 18Co fibroblasts in the presence of SRI 31215 (10 μM) or JNJ 38877605 (1 μM) as indicated. Images were taken 24 hours after treatment.

To demonstrate that SRI 31215 prevents fibroblast-induced MET activation, we treated serum-starved DU145 cells with conditioned media from pro-HGF-producing 18Co fibroblasts [48] or with recombinant, active, HGF. Both recombinant HGF and fibroblast-derived factors triggered activation of MET, GAB1, ERK, AKT and STAT3 in DU145 cells. The MET kinase inhibitor, JNJ 38877605, prevented HGF- and fibroblast-induced activation of MET and it's downstream signaling proteins (Figure 2A). In contrast, SRI 31215 prevented fibroblast-induced MET activation and signaling in tumor cells, but did not prevent MET activation induced by active HGF (Figure 2A). SRI 31215 inhibited fibroblast-induced ERK1/2, AKT and STAT3 activation in a dose-dependent manner (Supplementary Figure S2).

HGF was identified as a scatter factor for its ability to induce scattering of cancer cells [1, 49]. Indeed, we showed that both recombinant HGF and 18Co fibroblasts induce scattering of DU145 cells, an established model to study cell scattering [50]. While SRI 31215 did not interfere with scattering induced by active HGF, it prevented fibroblast-induced cell scattering as effectively as the kinase inhibitor JNJ 38877605 (Figure 2B). SRI 31215 blocked scattering of cancer cells in a dose-dependent manner with biological activity detected at 1μM [46].

These data demonstrate that SRI 31215, a triplex inhibitor of matriptase, hepsin and HGFA, blocks pro-HGF activation, prevents the crosstalk between tumor cells and tumor associated fibroblasts and inhibits fibroblast-induced oncogenic signaling in tumor cells.

### SRI 31215 inhibits fibroblast-induced epithelial mesenchymal transition (EMT) in tumor cells

**Figure 3:**
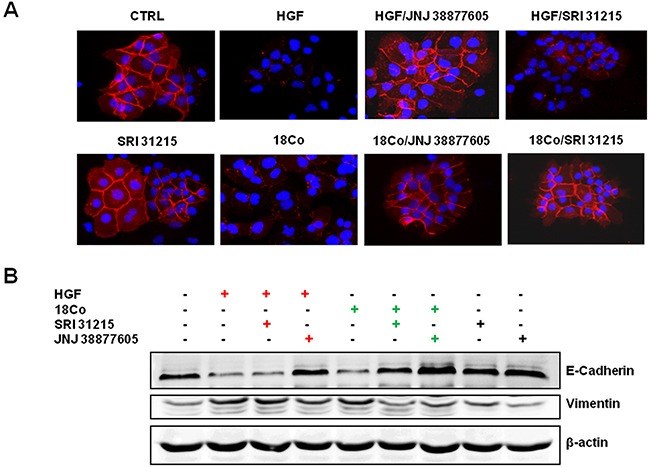
SRI 31215 inhibits fibroblast-induced EMT in DU145 cells Cells were stimulated with HGF or 18Co conditioned media for 24 hours in the absence or presence of SRI 31215 (10 μM) or JNJ 38877605 (1 μM) as indicated. The expression of E-cadherin was monitored by immunofluorescence **A.** The levels of E-cadherin and vimentin were also determined by immunoblotting **B.**

The scattering of epithelial cells is linked to the loss of epithelial cell-cell junctions and the acquisition of a motile mesenchymal cell phenotype, which are both hallmarks of the epithelial-mesenchymal transition (EMT). A crucial event during EMT is downregulation of E-cadherin, an epithelial marker, coupled to upregulation of vimentin, a marker of the mesenchymal phenotype. We demonstrated that both recombinant HGF and pro-HGF-producing fibroblasts inhibit the expression of E-cadherin in DU145 cells (Figure 3A and 3B). JNJ 38877605, but not SRI 31215, restored the expression of E-cadherin in HGF-treated DU145 cells. In contrast, both SRI 31215 and JNJ 38877605 prevented fibroblast-induced inhibition of E-cadherin (Figure 3A and 3B). Both HGF and fibroblasts induced the expression of vimentin in DU145 cells, confirming that they promote EMT in cancer cells. Both SRI 31215 and JNJ 38877605 prevented fibroblast-induced expression of vimentin (Figure 3B).

These data demonstrate that fibroblasts induce EMT in cancer cells in an HGF-dependent manner and that SRI 31215 inhibits fibroblast-induced EMT in cancer cells.

### SRI 31215 inhibits fibroblast-induced migration of cancer cells

**Figure 4:**
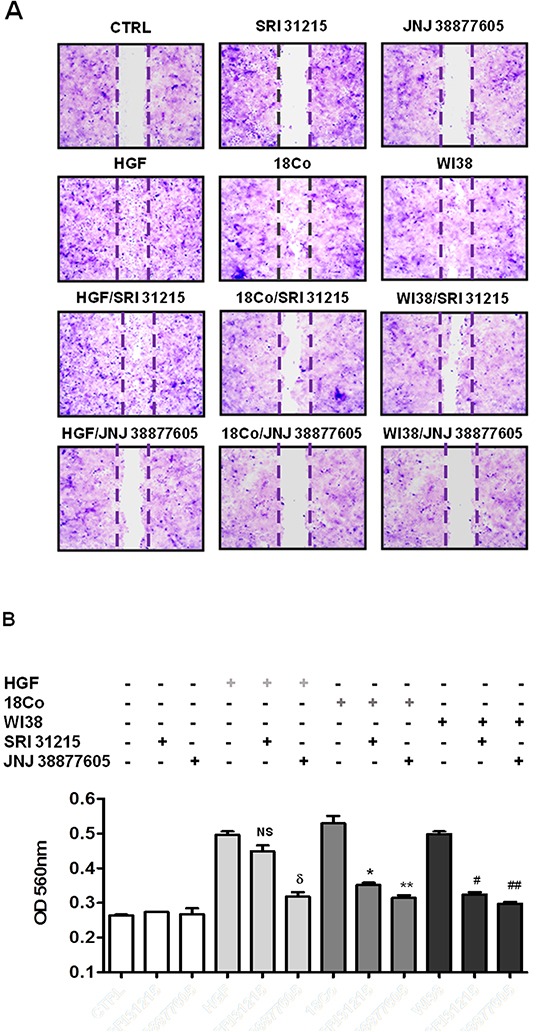
SRI 31215 inhibits the fibroblast-mediated migration of cancer cells **A.** Serum starved monolayers of DU145 cells were scratched and were left untreated or were treated with HGF or conditioned media from 18Co or WI38 fibroblasts in the absence or presence of SRI 31215 (10 μM) or JNJ 38877605 (1 μM) as indicated. Plates were stained with crystal violet 24 hours after treatment. **B.** Transmigration assay of DU145 cells was performed as described in Material and Methods. NS: not significant, δ: p<0.004, *:p<0.008, **:p<0.006, #:p<0.002, ##:p<0.001.

Epithelial mesenchymal transition is tightly linked to the migration and invasion of cancer cells. Indeed, using a scratch assay (Figure 4A) or a transwell migration assay (Figure 4B) we confirmed that recombinant HGF or pro-HGF-producing fibroblasts (18Co and WI38) stimulate the migration of DU145 cells. The MET kinase inhibitor, JNJ 38877605, completely prevented both HGF and fibroblast-induced migration of cancer cells (Figure 4A and 4B). SRI 31215 did not interfere with HGF-induced migration, but inhibited fibroblast-induced migration of DU145 cells as effectively as JNJ 38877605. We demonstrated that SRI 31215 and JNJ 38877605 also inhibit basal, constitutive, migration of RKO cells (data not shown), which produce HGF [12] and activate MET in an autocrine manner [51].

These results demonstrate that fibroblasts promote the migration of cancer cells in an HGF-dependent manner and that SRI 31215 blocks signaling between tumor cells and fibroblasts, inhibiting their tumor-promoting activity.

### SRI 31215 overcomes the resistance to EGFR inhibitors mediated by autocrine HGF/MET signaling in colon cancer cells

Inhibitors of EGFR (EGFRi), such as cetuximab and panitumumab, have been used successfully for the treatment of colon cancer patients with WT Kras [52–54]. However, only a subpopulation of patients that harbor WT KRas respond to this treatment and the mechanisms of primary resistance to EGFRi in these patients are not completely understood.

**Figure 5:**
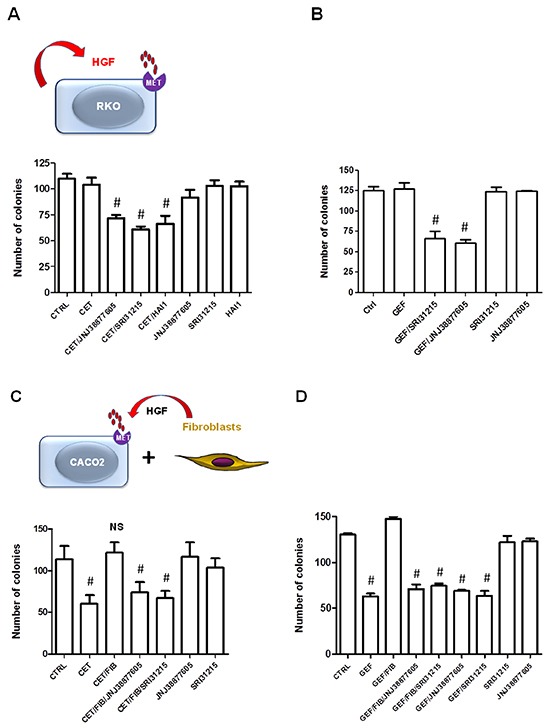
Inhibitors of HGF/MET signaling prevent the innate and acquired resistance to cetuximab **A.** and **B.** RKO cells were treated with cetuximab (50 μg/ml) (A) of gefitinib (B) alone, or in combination with SRI 31215 (10 μM), JNJ 38877605 (1 μM) or recombinant HAI-1 (20 nM) as indicated and clonogenic growth was monitored as described in Materials and Methods. **C.** and **D.** Caco2 cells were treated with cetuximab or gefitinib alone or in the presence of CM from fibroblasts and SRI 31215 (10 μM) or JNJ 38877605 (1 μM) as indicated. #: p<0.05, compared to control (CTRL); NS: not significant.

We tested the hypothesis that autocrine production of HGF by colon cancer cells confers resistance to EGFRi. We used HGF-producing RKO cells which, despite carrying WT KRas, do not respond to cetuximab and represent a large proportion of colon cancer patients with WT KRas that fail to respond to EGFRi. Treatment of RKO cells with SRI 31215, recombinant HAI or JNJ 38877605 alone did not impact the clonogenic growth of these cells, demonstrating that these cells are not addicted to autocrine HGF/MET signaling. However, when we treated RKO cells with a combination of cetuximab and either SRI 31215, JNJ 38877605 or recombinant HAI-1, we observed a significant decrease in their clonogenic growth (Figure 5A). Both SRI 31215 and JNJ 38877605 also sensitized HGF-producing RKO cells to gefitinib (Figure 5B, Supplementary Figure S3A). These data demonstrate that primary resistance to EGFRi may be due to the autocrine production of HGF, which has been recently shown to occur in approximately 30% of colon cancers due to mutations in the HGF promoter region [12]. Similar results were found in HCT116 cells, which, like RKO cells, produce HGF [12], but carry MT KRas (data not shown). In contrast, JNJ 388777605 or SRI 31215 did not improve the response to EGFRi in HT29 cells (Supplementary Figure S3B) which do not make HGF [12].

Our findings show that overproduction of HGF can cause primary resistance to EGFRi in colon cancer cells and suggest that such patients would benefit from combined therapy with inhibitors of EGFR and HGF. Indeed, high serum levels of HGF have recently been shown to be associated with resistance to EGFRi therapy in colon cancer patients with WT KRas [55].

### SRI 31215 overcomes fibroblast-mediated resistance of colon cancer cells to EGFR inhibitors

Resistance to targeted therapy can also originate from the tumor microenvironment where fibroblasts are a common source of pro-HGF [9, 56, 57]. We demonstrated that conditioned medium (CM) from 18Co fibroblasts inhibits the response of Caco2 cells to cetuximab. Both SRI 31215 and JNJ 38877605 restored the response of Caco2 cells to cetuximab in the presence of fibroblast-derived CM (Figure 5C), demonstrating that fibroblasts inhibit the response to cetuximab through secretion of pro-HGF. We confirmed that Caco-2 cells failed to respond to gefitinib when we co-cultured with 18Co fibroblasts (data not shown).

Caco2 cells also failed to respond to the EGFR kinase inhibitor gefitinib when exposed to conditioned medium from 18Co fibroblasts (Figure 5D). Treatment of cancer cells with SRI 31215 or JNJ 38877605 overcame fibroblast-mediated resistance to gefitinib (Figure 5D).

Together these data demonstrate that SRI31215 inhibits autocrine or paracrine HGF/MET signaling and thus averts the resistance of colon cancer cells to EGFRi.

### SRI31215 averts fibroblast-mediated resistance to EGFRi-induced apoptosis

**Figure 6:**
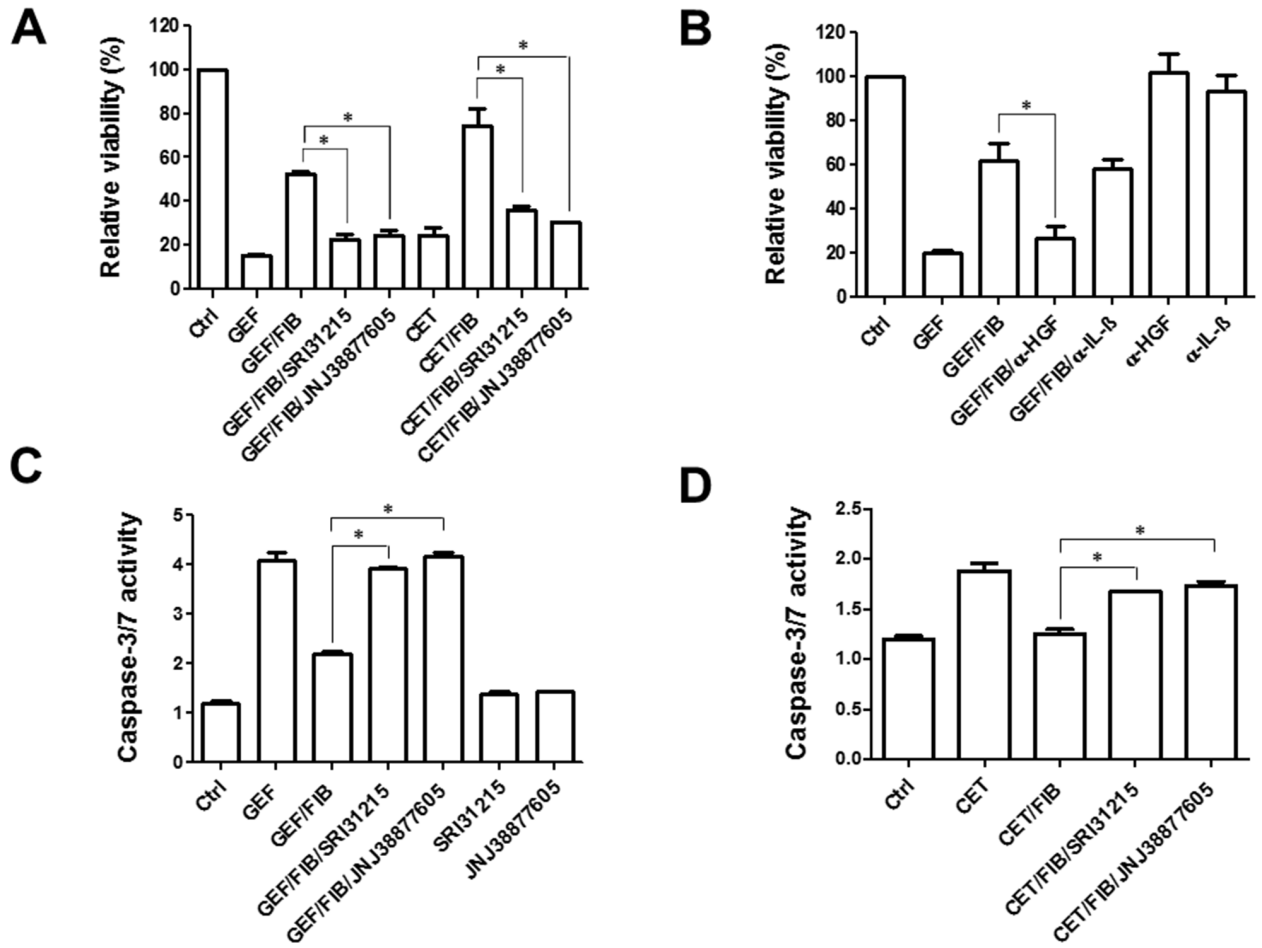
Fibroblasts inhibit the proapoptotic activity of EGFRi in an HGF-dependent manner **A.** and **B.** DiFi cells were treated with gefitinib (0.5 μM) or cetuximab (0.5 μg/ml) in the absence or the presence of conditioned medium from fibroblasts and SRI 31215 and JNJ 38877605 as indicated. The viability was determined after 72 hours. In B, fibroblast conditioned medium was incubated with anti HGF or anti-IL1β antibodies. **C.** and **D.** DiFi cells were treated with gefitinib (C) or cetuximab (D) in the presence of conditioned medium from fibroblasts and inhibitors of HGF/MET signaling as indicated for 18h. Caspase activity was assessed by the Caspase-Glo 3/7 Assay from Promega.

DiFi cells undergo apoptosis in response to inhibition of EGFR [58]. Fibroblasts inhibited the response of DiFi cells to both gefitinib and cetuximab (Figure 6). We demonstrated that both SRI 31215 and JNJ 38877605 prevent fibroblast- mediated resistance to gefitinib and cetuximab (Figure 6A), suggesting that fibroblasts inhibit the response to EGFRi through HGF. Indeed, we demonstrated that HGF is sufficient to protect DiFi cells from EGFRi (Supplementary Figure S4). HGF is required for the prosurvival activity of fibroblasts as antibody-based neutralization of HGF abrogates the prosurvival activity of fibroblasts (Figure 6B). In contrast, neutralization of IL-1β did not impact the ability of fibroblast to protect DiFi cells from gefitinib (Figure 6B). We showed that gefitinib-induced activation of caspase 3 and caspase 7 was inhibited by fibroblasts and restored by SRI 31215 or JNJ 38877605 (Figure 6C). Similar results were obtained with cetuximab (Figure 6D).

**Figure 7:**
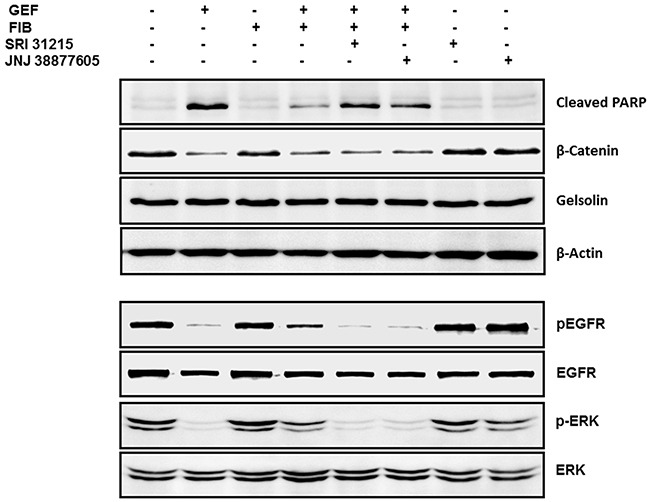
Fibroblasts protect DiFi cells from EGFRi in an HGF-dependent manner DiFi cells were treated with gefitinib (0.5 μM) alone or were co-cultured with WI38 fibroblasts (FIB) in the absence or the presence of SRI 31215 (10 μM) or JNJ38877605 (1 μM). The DiFi cell lysates were isolated after 4 or 24 hours as indicated and examined for the expression of pERK and total ERK, pEGFR and total EGFR, cleaved PARP, β-catenin gelsolin and β-actin by immunoblotting.

Fibroblasts interfered with gefitinib-induced cleavage of PARP, confirming that they inhibit gefitinib-induced apoptosis. SRI31215 or JNJ 38877605 restored gefitinib-induced cleavage of PARP in the presence of fibroblasts, demonstrating that fibroblasts interfere with gefitinib-induced apoptosis through HGF. Gefitinib also reduced the levels of β-catenin in cancer cells, however neither fibroblasts nor inhibitors of HGF/MET signaling had any effect on β-catenin levels in DiFi cells. The levels of gelsolin or β-actin were not altered by either gefitinib or fibroblasts (Figure 7).

Fibroblasts interfered with gefitinib-induced inhibition of EGFR and ERK phosphorylation. When DiFi cells were co-cultured with fibroblasts, the cancer cells maintained phosphorylation of EGFR and ERK1/2 despite treatment with gefitinib (Figure 7). The levels of total ERK or total EGFR were not modulated by fibroblasts or by gefitinib. Inhibition of HGF/MET signaling by SRI 31215 or by the MET kinase inhibitor JNJ 38877605 blocked the prosurvival activity of fibroblasts (Figure 7), confirming that fibroblasts elicit resistance to gefitinib in an HGF-dependent manner.

## DISCUSSION

Aberrant HGF/MET signaling supports cell survival, proliferation, angiogenesis, invasion and metastatic spread, which are essential hallmarks of cancer. Several cancers are addicted to HGF/MET signaling, establishing both HGF and MET as valid therapeutic targets. Indeed, neutralizing antibodies targeting MET or HGF and a large number of MET kinase inhibitors have entered clinical trials, yielding encouraging results [4, 11, 59].

HGF is synthesized and secreted by tumor cells or by stromal cells, such as fibroblasts, as the inactive precursor, pro-HGF. The proteolytic conversion of pro-HGF to its active form is the rate-limiting step in the HGF/MET signaling pathway. Activation of pro-HGF is primarily driven by matriptase, hepsin and HGFA [30], serine proteases that are commonly overexpressed in cancer tissues. The activity of matriptase, hepsin and HGFA is regulated by HAI-1/2 [30, 60], which act as endogenous inhibitors of HGF activation. Intestinal deletion of endogenous HAI-1 augments Wnt signaling in Apc/^Min/+^ mice, both in tumors and in normal mucosa and enhances Apc-initiated tumor formation [34], suggesting that HAI-1 has tumor suppressor properties. Indeed, reduced expression of HAIs is associated with advanced disease and poor outcome in cancer patients [34–40].

We synthesized SRI 31215, a small molecule that acts as a triplex inhibitor of matriptase, hepsin, and HGFA, mimicking the activity of HAI-1 [46]. Here we showed that SRI 31215 inhibits activation of pro-HGF and thereby blocks HGF-dependent MET signaling. Recently Han et al. reported development of peptidylketothiazole inhibitors of matriptase, hepsin and HGFA as a nonkinase strategy to inhibit HGF/MET signaling in cancer cells [61]. Inhibitors of HGF activation, such as SRI 31215, not only limit the amount of biologically active HGF, but also result in the accumulation of pro-HGF, which acts as a receptor antagonist [62]. Indeed, the expression of ‘uncleavable’ pro-HGF (generated by mutating the cleavage site at Arg494/Val495 to Asp494/Val495) prevents tumor growth *in vivo* and metastatic spread of cancer cells [62].

HGF has recently been shown to be produced in a relatively large subset (~30%) of primary colon tumors and established colon cancer cell lines due to mutations in the HGF promoter region [12]. Here we show that inhibition of HGF activation by SRI 31215 or inhibition of MET kinase activity sensitize HGF-producing RKO cells to cetuximab and gefitinib. Although EGFRi are approved for the treatment of metastatic colon cancer patients with WT KRas, only 17% of KRas WT patients benefit from panitumumab [63] and only 12.8% of the patients respond to cetuximab [64]. Our findings demonstrate that overproduction of HGF can underlie primary resistance to EGFRi in colon cancer cells that harbor WT KRas and suggest that these patients may benefit from combined therapy with inhibitors of EGFR and HGF. Indeed, high serum levels of HGF have recently been shown to be associated with resistance to EGFRi therapy in colon cancer patients with WT KRas [55]. Thus, serum levels of HGF may constitute a simple strategy to select patients for targeted anti-HGF therapy.

Cancer-associated fibroblasts promote malignant cell growth and survival at least in part by HGF secretion [65]. We confirmed that HGF is sufficient to promote the growth and survival of colon cancer cells and that antibody-mediated neutralization of HGF abrogates the tumor-promoting activity of fibroblasts (Supplementary Figure S1, Figure 6B). Both the MET kinase inhibitor JNJ38877605 and SRI 31215 inhibit signaling between cancer cells and HGF-producing fibroblasts, blocking fibroblast-induced proliferation, EMT and migration of cancer cells. We confirmed that structurally distinct triplex inhibitors of matriptase, hepsin and HGFA block the crosstalk between tumor cells and fibroblasts [66]. Furthermore, we demonstrated that SRI 31215 overcomes fibroblast-mediated resistance to EGFR inhibitors in colon cancer cells. Thus, inhibitors of HGF activation, such as SRI 31215, represent a novel approach to block the crosstalk between tumor cells and fibroblasts, neutralizing the tumor-promoting activity of cancer-associated fibroblasts.

Our data established that SRI 31215 inhibits ligand-induced MET activation. However, because the transforming potential of mutant MET appears to depend on the presence of HGF [67], it is possible that inhibitors of HGF activation may block tumor progression in cancers that are driven by mutant MET. Furthermore, recent data strongly suggest that an effective blockade of HGF/MET signaling requires simultaneous inhibition of both the receptor and the ligand. For example, it has been demonstrated that lung cancer cells with amplified MET become dependent on HGF under pharmacological MET inhibition [10]. Similarly, although MET kinase inhibitors curb the growth of leukemic cells that are addicted to HGF/MET signaling [13], cancer cells rapidly develop resistance to MET kinase inhibitors due to compensatory upregulation of HGF. It therefore appears that concurrent inhibition of MET and HGF may be required to overcome resistance to MET kinase inhibitors.

Preclinical studies have provided strong support for the notion that inhibitors of HGF/MET signaling have therapeutic efficacy in a selected group of cancer patients. We demonstrated that inhibitors of HGF activation such as SRI 31215 represent a novel approach to inhibit autocrine and paracrine oncogenic HGF/MET signaling and to prevent HGF-dependent proliferation, EMT and migration of cancer cells. In addition, our study indicates that dual inhibition of HGF and EGFR precludes primary and acquired, fibroblast-mediated, resistance to EGFRi in colon cancer cells. Mechanism-based combination therapy is crucial to obtain sustained remission and to improve the outcome of colorectal cancer patients.

## MATERIALS AND METHODS

### Synthesis of SRI 31215

SRI 31215, the hydrogen chloride salt of 3-(3- ((1-benzylpiperidin-4-yl)methyl)-5-methyl-2-oxotetrahydropyrimidin-1(2H)-yl)benzimidamide, was developed at Southern Research. SRI 31215 was synthesized in six reaction steps with a 6% overall yield from readily available starting materials. Full experimental details on the synthesis of SRI 31215 have been published elsewhere [46].

### Protease panel

The inhibitory activity of SRI 31215 was tested in a panel of six proteases, which included matriptase, hepsin, HGFA, trypsin, thrombin and coagulation factor Xa. All six enzymes were purchased from R&D Systems. The assay buffer used was 50 mM Tris, 20 mM NaCl, 0.01% Tween 20, pH 8.0. The substrate was a custom FRET peptide based on the pro-HGF cleavage sequence (H2N-(EEdans)GKQLRVVNGG(KDabcyl)-amide) pre- pared by New England Peptide. All measurements were made using an Aminco-Bowman Series 2 Luminescence Spectrometer.

### Cell culture

DU145 cells were maintained in RPMI and Caco2, RKO, 18Co and WI38 cells in minimum essential medium (MEM), supplemented with 10% fetal bovine serum, L-glutamine and antibiotics under standard cell culture conditions. Conditioned medium from fibroblasts was prepared as follows: 18Co or WI38 fibroblasts were maintained in complete medium until they reached confluence. Confluent cultures were briefly rinsed and maintained in serum free MEM medium for another 36 hours. To ensure that conditioned medium did not contain active HGF, in some experiments fibroblasts were pre-treated with SRI 31215. Cell supernatants were collected, centrifuged and used immediately or they were aliquoted and stored at −80°C.

### Proteolytic activation of pro-HGF

Recombinant human HAI-1, recombinant human HGFA, and recombinant human HGF propeptide (pro-HGF) were purchased from R&D Systems. SRI 31215 (10 μM) or recombinant human HAI-1 (20 nM) were mixed with activated recombinant human HGFA pro-peptide (1nM) in TNC buffer (pH 8.0) and incubated at room temperature for 30 minutes. Recombinant human pro-HGF (40 ng) was added and incubated at 37°C for 1 hour. The reaction was stopped by SDS-PAGE gel sample buffer and samples were boiled and separated by 12% PAGE. Proteins were transferred onto nitrocellulose membrane, blocked with 5% milk and immunoblotted using antibodies that recognize pro-HGF as well as α and β chains of activated HGF.

### Cell scattering assay

DU145 cells were cultured in 6-well tissue culture plates at a density of 1×10^3^ cells per well. After colonies formed (6-8 days), cells were serum-starved overnight and were then treated with recombinant HGF (10 ng/ml) or with conditioned media from 18Co fibroblasts in the presence or absence of SRI 31215 (10 μM) or the MET kinase inhibitor JNJ 38877605 (1 μM) for 24 hours. Cells were washed with PBS and colonies were fixed and stained with 0.5% crystal violet solution in 6% glutaraldehyde.

### Wound healing/scratch assay

Cancer cells were seeded in 12-well plates and allowed to reach confluence. Following overnight serum-starvation, a scratch/wound was introduced into the cell monolayer with a sterile tip. Cells were cultured in serum-free media or were treated with recombinant HGF (10 ng/ml) or serum-free conditioned media from fibroblasts (50%) in the presence or absence of SRI 31215 or JNJ 38877605. Images of migrating cells were captured at 0, 12 and 24 hours after the treatment. Colonies of cells were stained with crystal violet for better visualization as shown in Figure 4A.

### Transwell migration assay

Cytoselect cell migration assay kit was purchased from Cell Biolabs, Inc and the assay was performed according to the manufacturer's instructions. Briefly, DU145 cells (75×10^3^) were seeded in transwells (8 μM pore size inserts) in serum-free media and were stimulated with recombinant HGF or with conditioned media from 18Co or WI38 fibroblasts (in the absence or the presence of SRI 31215 or JNJ 38877605). Cells were incubated for 12 hours and non-migratory cells were gently removed from inside of the inserts. Inserts were stained for 10 minutes at room temperature, transferred to wells containing 200 μl of extraction buffer and incubated for 10 minutes on a shaker. The optical density (OD) was measured at 560 nm.

### Viability and clonogenic assays

Cell titer-glo luminescent cell viability assay kit was purchased from Promega. Cells were seeded in 96-well plates at a density of 1×10^4^ cells per well and cell viability was assessed after the indicated time points as suggested by the manufacturer. For clonogenic assay, cells were seeded in 6-well plates at a density of 400 cells per well, serum starved overnight, and treated as indicated. After colony formation (8-12 days), cells were stained with crystal violet and the number of colonies was determined using ImageQuantTL (GE Health Care Life Sciences).

### Western blotting

Immunoblotting was performed using standard procedures. Membranes were blocked with 5% non-fat milk for 1 hour at RT, and incubated with primary antibodies overnight at 4°C. Primary antibodies used were anti-HGF (R&D Systems), anti-MET, anti-p-MET, anti-p-GAB1, anti-p-AKT, anti-p-ERK1/2, anti-vimentin (Cell Signaling Technology), anti-p-STAT3 (Upstate Cell Signaling Solutions), anti-E-cadherin (BD Transduction Laboratories), anti-β-actin (Sigma). Following washing with TBS-T buffer, membranes were incubated with horseradish peroxidase-conjugated secondary antibodies for 1 hour and then washed with TBS-T. Immunoblots were developed using the chemiluminescent detection system with ECL (Amersham). Protein loading was normalized by probing blots for the expression of β-actin.

### Immunofluorescence

Cells were seeded at 1×10^3^ cells per well in chamber slides. Following overnight serum-starvation, cells were treated with recombinant HGF (10 ng/ml) or conditioned media prepared from fibroblasts (50%) in the presence or absence of SRI 31215 (10 μM) or JNJ 38877605 (1 μM) for 24 hours. Cells were washed with PBS, fixed in ice-cold methanol/acetic acid solution [95:5 (v/v)] for 20 minutes at-20°C and incubated with anti-E-cadherin antibodies overnight at 4°C. Slides were washed with PBS, incubated with Alexa Fluor 568 conjugated secondary antibody for 1 hour at 37°C and examined with a fluorescent microscope.

### Caspase3/7 activity

DiFi cells were seeded at a density of 20,000 cells/well in a 96 well plate and were treated with EGFRi in the absence or the presence of SRI 31215 or JNJ 38877605 for 18h. Caspase activity was assessed by the Caspase-Glo 3/7 Assay from Promega following the manufacturer's instructions.

### Statistical analyses

All experiments were repeated at least three times. Values in Figure 1 to Figure 6 are given as the mean ± SEM. Statistical analyses were performed using GraphPad Prism 5.0 (Student's t-test) and values with *p* <0.05 were considered statistically significant.

## SUPPLEMENTARY FIGURES


