# Supplementary material for: Inhibition of pro-HGF activation by SRI31215, a novel approach to block oncogenic HGF/MET signaling
Source: Oncotarget. 2016 Apr 18;7(20):29492–506. doi: 10.18632/oncotarget.8785 (PMC5045412; doi:10.18632/oncotarget.8785)
Supplement: Supplementary file 1 [file oncotarget-07-29492-s001.pdf]

SUPPLEMENTARY FIGURES

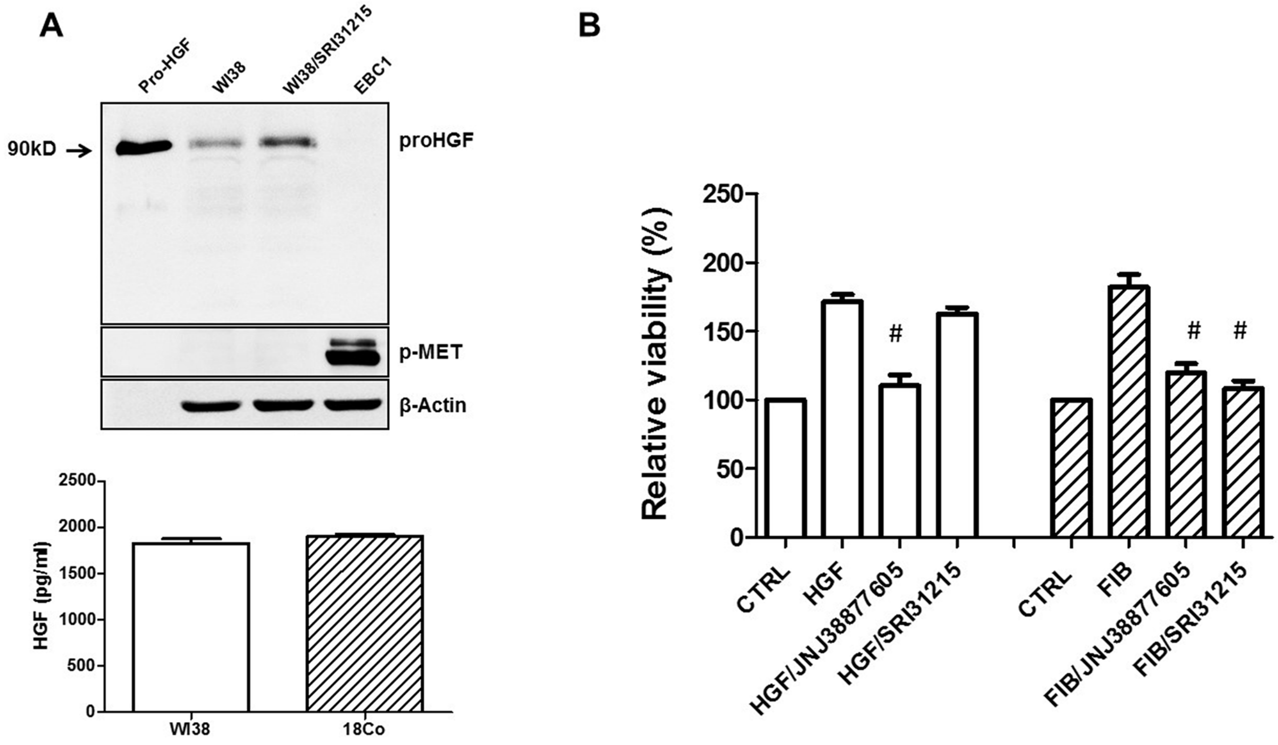

Supplementary Figure S1: A. The expression of pro-HGF and pMET in WI38 fibroblasts as determined by immunoblotting, and ELISA in WI38 and 18Co fibroblasts. Cell lysates from EBC cells were used as control for pMET. **B.** DiFi cells were cultured in the presence of HGF or fibroblast conditioned medium in the absence or the presence of JNJ38877605 or SRI 31215 for 48 hours. Neutralization of HGF in fibroblast conditioned medium by anti-HGF antibodies inhibits growth promoting activity of fibroblasts (data not shown).

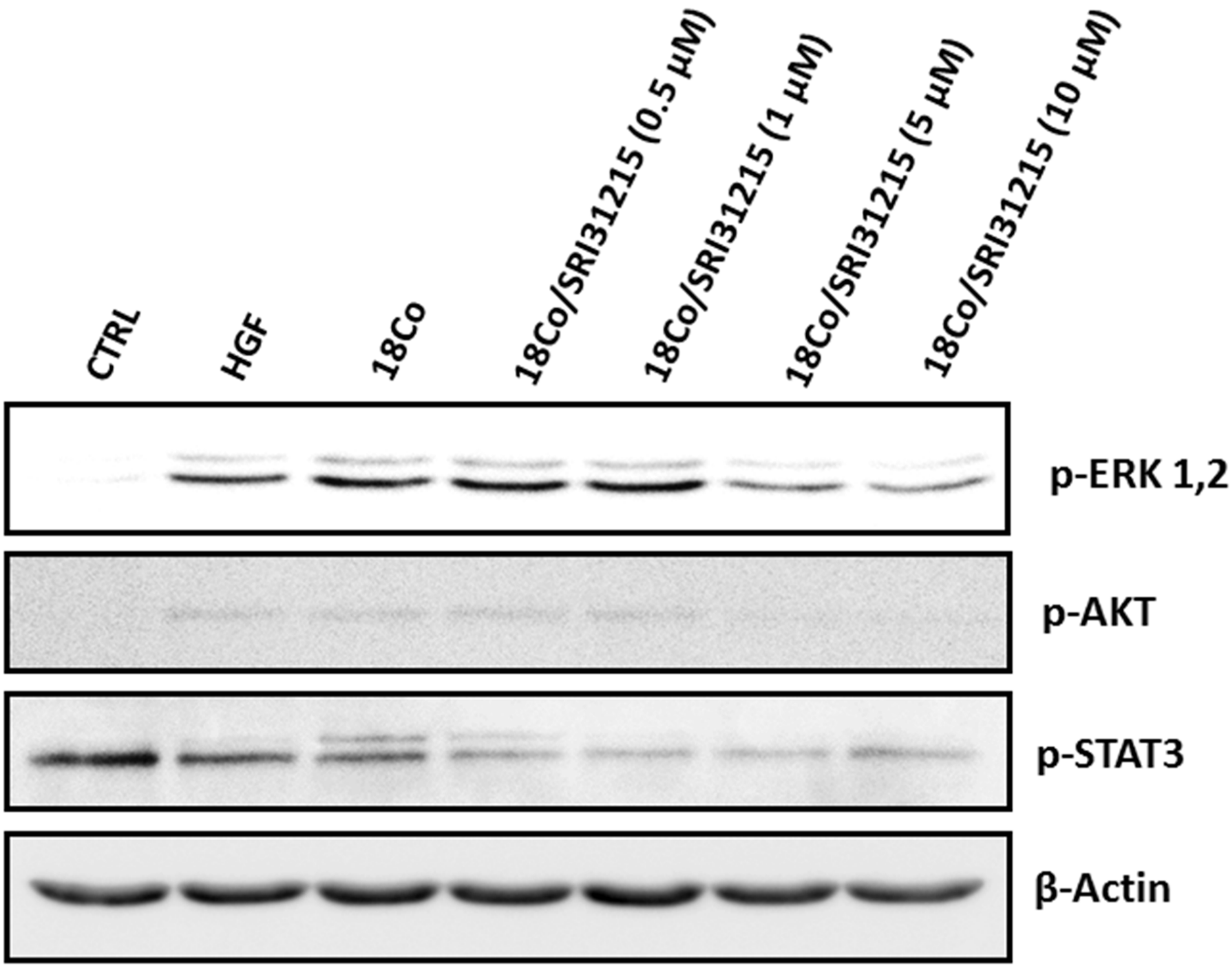

Supplementary Figure S2: Dose-dependent inhibition of fibroblast induced MET activation by SRI 31215. DU145 cells were serum-starved and were treated with conditioned medium from 18Co fibroblasts for 30 minutes in the absence or the presence of increasing concentrations of SRI 31215 as indicated. The levels of pERK1/2, pAKT, pSTAT3 and β-actin were determined by immunoblotting.

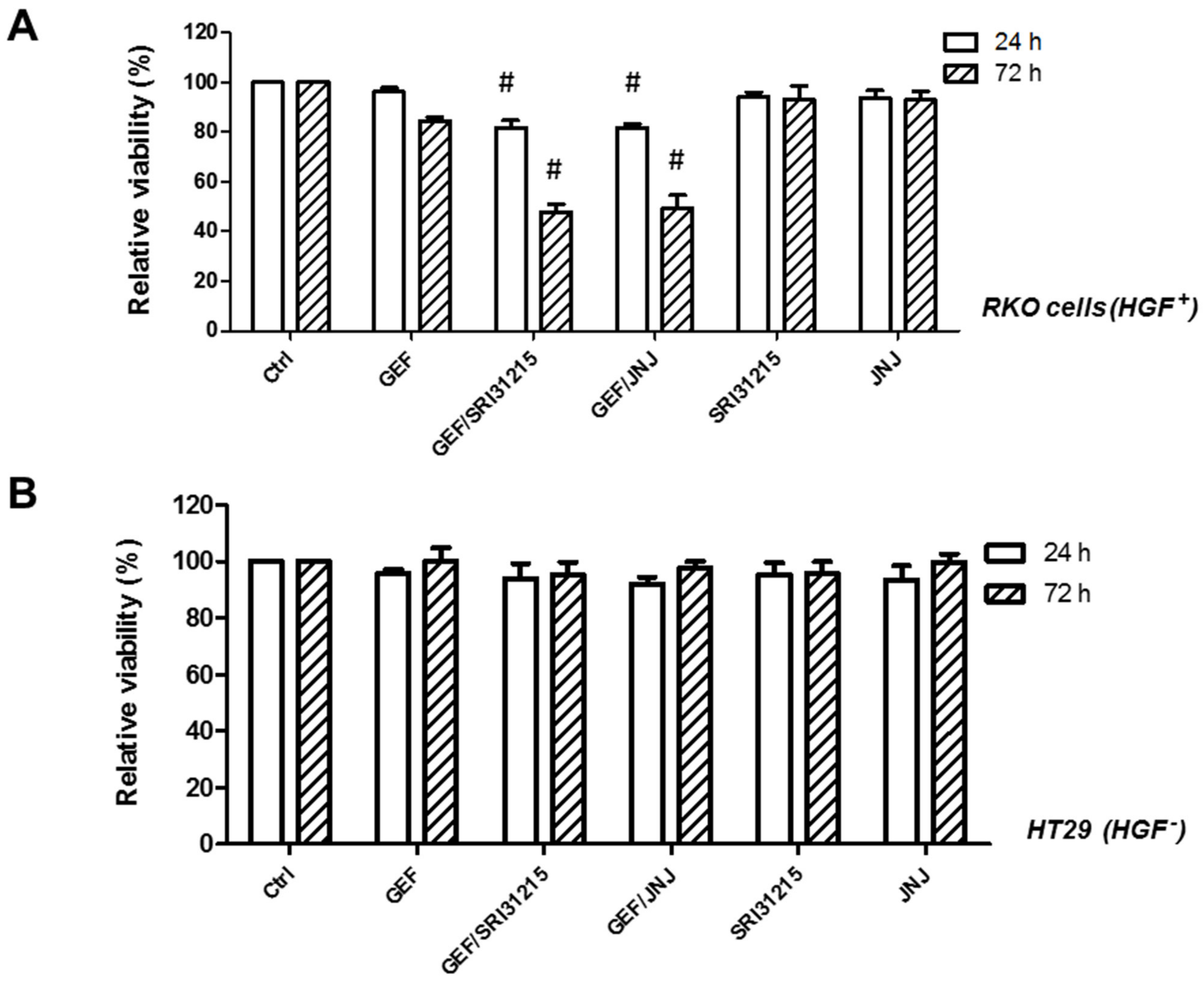

Supplementary Figure S3: Inhibition of HGF/MET signaling sensitizes HGF-producing RKO colon cancer cells to gefitinib. A. RKO cells (HGF^+) and **B.** HT29 (HGF^-) cells were treated with gefitinib (5 µM) in the absence or the presence of SRI 31215 (10 µM) or JNJ 38877605 (1 µM) as indicated and viability was determined 24 and 72 hours after treatment. # indicates statistical significance compared to samples treated with gefitinib alone as determined by Students' t-test (*p*<0.05).

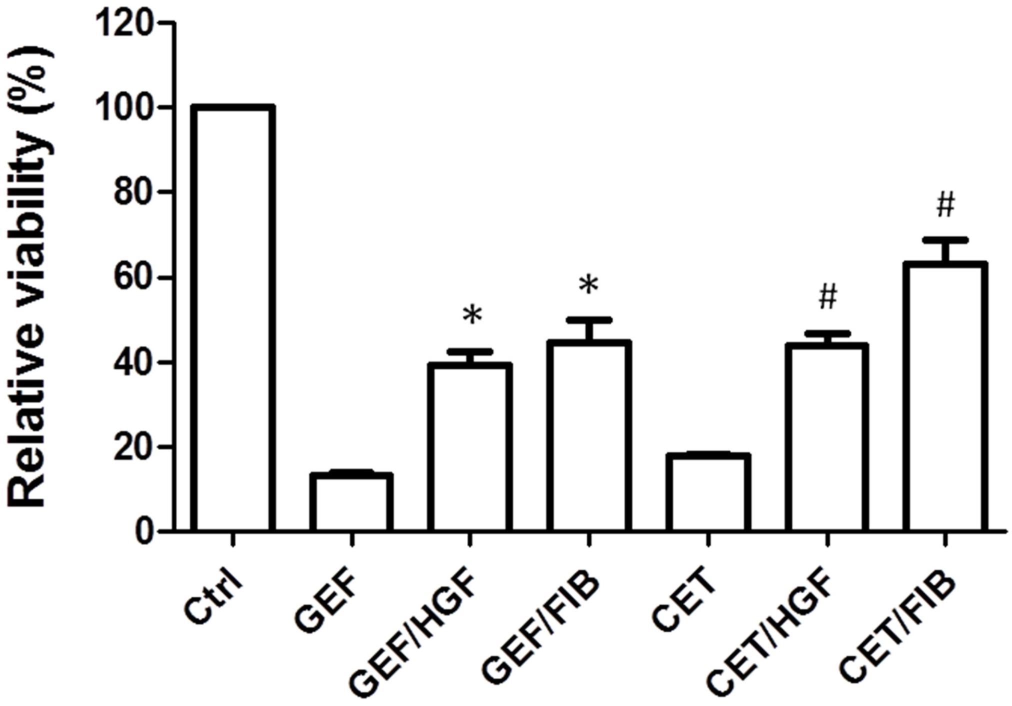

Supplementary Figure S4: HGF is sufficient to protect DiFI cells from gefitinib or cetuximab-induced cell death. Cells were treated with gefitinib (GEF) or cetuximab (CET) in the absence or the presence of HGF or fibroblast (FIB)-derived factors as indicated and the viability was determined after 72 hours.
